# Embolic Stroke as the Initial Manifestation of Systemic Lupus Erythematosus

**DOI:** 10.1155/2015/373201

**Published:** 2015-07-22

**Authors:** Reshma M. Khan, Rajaie Namas, Sachin Parikh, Bernard Rubin

**Affiliations:** ^1^Department of Internal Medicine, Division of Rheumatology, Henry Ford Hospital, Wayne State University, Detroit, MI 48202, USA; ^2^Department of Internal Medicine, Division of Rheumatology, University of Michigan, Ann Arbor, MI 48109, USA; ^3^Department of Internal Medicine, Division of Cardiology, Henry Ford Hospital, Detroit, MI 48202, USA

## Abstract

We present a case of a 21-year-old African-American female with no significant medical history, who presented to the emergency department with a one-week history of blurry and double vision. Ophthalmology evaluation revealed bilateral retinal artery occlusion. Further workup with imaging of the brain was consistent with an ischemic stroke. Hereditary hypercoagulable workup was unremarkable and initial testing for antiphospholipid syndrome was positive. She underwent transesophageal echocardiogram (TEE), which showed severe mitral regurgitation and thickening of mitral valve leaflets consistent with Libman-Sacks endocarditis. Autoimmune workup was positive for IF-ANA, anti-RNP, and anti-Smith antibody. She fulfilled 4/11 of the ACR criteria and met 5 of the SLICC (Systemic Lupus International Collaborating Clinics) criteria for lupus (nonscaring alopecia, thrombocytopenia, positive ANA, and positive anti-Smith and positive anti-phospholipid antibodies). This case highlights the importance of early recognition of underlying connective tissue diseases and timely management of these diseases in young patients with no previous manifestations of diseases.

## 1. Case Report

A previously healthy 21-year-old African-American female was transferred to our hospital for escalation of treatment. She presented to the emergency department one week prior to this admission with progressive worsening of the vision in the right eye described as double vision associated with pain and redness. Approximately over the last three weeks prior to this admission she was experiencing excruciating headaches associated with her eye symptoms. She was evaluated at her primary-care physician's office and was found to have an elevated blood pressure and a new heart murmur. Review of systems was positive for unintentional weight gain of 13.5 kilograms in the last eight months, excessive fatigue, fever, difficulty focusing, one episode of painful oral ulcer on the roof of her mouth, change in the hair texture with nonscarring alopecia, shortness of breath with exertion, and constipation. She reports no history of malar rash, photosensitivity, arthralgia, morning stiffness, thromboembolic diseases, or miscarriages. Her family history was significant for systemic lupus in her mother's cousin. She was smoking 0.25 PPD, consumed alcohol socially, and denied any illicit drug abuse. She was sexually active with men and used condom for contraception. Her home medications included as needed Motrin and tramadol.

On physical exam, she was awake, lethargic, and afebrile with a pulse of 90 beats per minute, blood pressure of 180/95 millimeters of mercury, and a BMI of 26.58 kg/m^2^. Ophthalmic exam revealed a right eye lid ptosis, down and out position, and mydriasis, consistent with third nerve palsy. Her visual field exam revealed a right visual field defect with total superior temporal, inferior temporal, superior nasal, and inferior nasal deficiencies and left visual field defect with total inferior temporal and inferior nasal deficiencies. Cardiovascular exam was positive for III/VI holosystolic murmur at the apex and significant pitting edema in her both lower extremities. Her musculoskeletal exam was unremarkable. Skin exam showed a diffuse macular hyper pigmentation of her back and a small-healed blister over the right shoulder. Her performance on a brief cognitive screen was remarkable for difficulties with basic attention, motor sequencing, impulse control, and retrieval of information (Cog Log Score 11/30).

**Figure 1:**
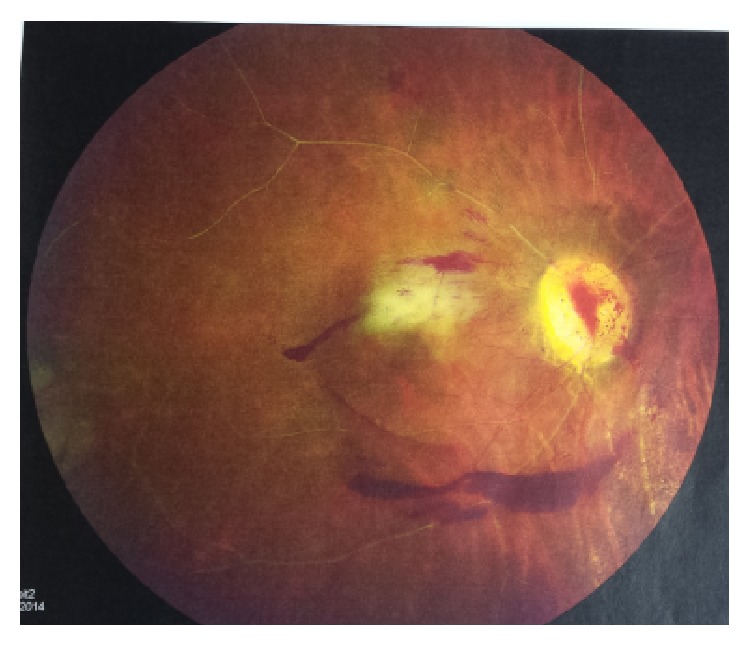
A fundoscopic exam of the right eye revealing vitreal hemorrhage and central retinal artery occlusion.

**Figure 2:**
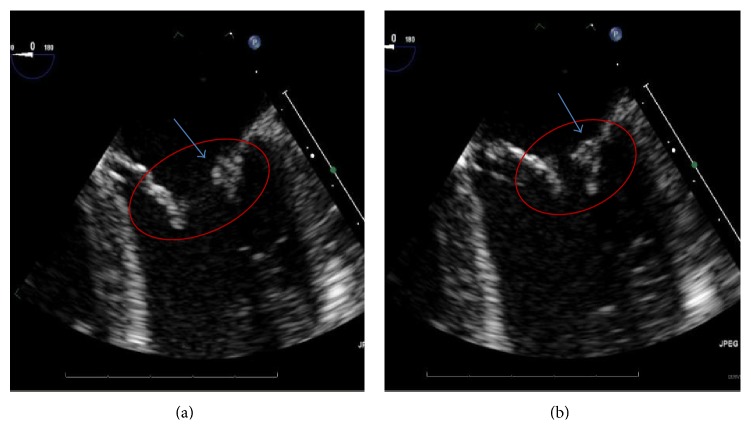
((a) and (b)) A cardiac transesophageal echocardiogram revealing severe mitral regurgitation and thickening of mitral valve leaflets with mitral valve leaflets not coapting and a small mass on the atrial side of the anterior mitral valve leaflet (arrow) suggesting Libman-Sacks endocarditis.

The brain CT scan findings in the emergency department were consistent with an acute to subacute infarct in the posteroparietal/occipital lobe region, and the follow-up MRI showed scattered areas of restricted diffusion, suggestive of infarcts in the bilateral cerebral and cerebellar hemispheres from a likely embolic source, and focal chronic encephalomalacia changes were seen involving the right temporal-occipital lobe. MRA and MRV of brain and neck were unremarkable with no significant stenosis. Ophthalmology evaluation was positive for bilateral central retinal artery occlusion and vitreal hemorrhage (Figure 1). She underwent a TEE that showed moderate to severe mitral regurgitation with mild mitral valve thickening, mitral valve leaflets not coapting, and a small mass on the atrial side of the anterior mitral valve leaflet possibly representing old vegetation (Figure 2). The concern was raised for possible Libman-Sacks endocarditis versus infective endocarditis. Hereditary coagulopathy workup and infectious workups including three series of blood cultures, hepatitis panel, HIV screen, and Fluorescent Treponemal Antibody Absorption Test (FTA-ABS) were negative. Workup for antiphospholipid syndrome revealed positive lupus anticoagulant panel (high prothrombin time, high PTT, and high DRVVT), low titer cardiolipin IgG 23.7 (<15: absent, 15–19: inconclusive, 20–79: moderate positive, and ≥80: high positive), and negative beta 2 glycoprotein.

The autoimmune workup was remarkable for elevated inflammatory markers, positive IF-ANA 1 : 640 (homogenous pattern), positive RNP antibody 37 (ref. <20), and positive Smith antibody 34 (ref. <20). She had normal complements, negative DNA antibody screen, and negative SSA and SSB antibodies. Her urine drug screen and pregnancy tests were negative. Analysis of her urine showed +2 proteins and +3 bacteria; however, no red blood cells or casts were present. Her initial EKG showed normal sinus rhythm, normal axis, normal intervals, and no ST/T wave changes; chest X-ray showed cardiac enlargement with no acute pulmonary process, and ultrasound of her kidney showed unremarkable evaluation of the kidneys.

The patient laboratory result at the initial presentation, at presentation to our hospital, and on discharge is shown in Table 1.

In view of the unremarkable extensive infectious workup, hereditary coagulopathy workup, and clinical presentation accompanied by a positive lupus anticoagulant and lupus serology, coupled with TEE images, she was diagnosed with Libman-Sacks endocarditis attributed to aPS and SLE. She was started on intravenous heparin, which was bridged with oral warfarin to achieve an INR goal of 2-3. She was also started on 20 mg of oral prednisone daily in view of her symptoms related to her diagnosis of SLE. Her condition was stable throughout her hospital course except for elevated blood pressure. She did not require renal biopsy as her urine analysis did not show any dysmorphic red blood cells or cast, and her renal function gradually normalized. Her vision failed to improve significantly even after repeated laser photocoagulation treatment performed by ophthalmology team. She was discharged home on aspirin 81 mg and warfarin with INR goal 2-3 and 20 mg of oral prednisone daily. She eventually underwent mitral valve repair. Mitral valve biopsy revealed a cardiac valve with a collagenized fibrous nodule. It was planned to start her on hydroxychloroquine during her follow-up in the rheumatology clinic. However, ophthalmology team had concerns for hydroxychloroquine due to her retinal damage. She was started on mycophenolate mofetil as a steroid sparring agent. Currently, her clinical condition has been stable with mycophenolate mofetil, and she could taper prednisone to 5 mg daily without any relapse. Her diagnosis of aPS was confirmed by repeating anti-phospholipid antibodies on follow-up visit 16 weeks apart.

## 2. Discussion

SLE is a complex multisystem autoimmune disease which is diagnosed on the clinical basis supported by an autoimmune laboratory profile. In 1997, revised criterion to guide the diagnosis of SLE was established by the American College of Rheumatology (ACR) [1] that requires a serial or simultaneous presentation of four or more of the listed symptoms for the diagnosis of lupus, namely, malar rash, discoid rash, photosensitivity, oral ulcers, arthritis, serositis, renal disorder, neurologic disorder, hematologic disorder, immunologic disorder, and antinuclear antibody. Though the present case did not fulfill the ACR criteria at presentation, clinical diagnosis of SLE was advocated in the presence of mitral valve vegetation in an otherwise healthy lady with nonscaring alopecia, thrombocytopenia, and positive IF-ANA, anti-Smith, and anti-phospholipid antibodies. Interestingly anti-dsDNA level was within normal range in our patient. However this has been shown to be highly specific for SLE but is only present in 70% of cases.

In 1924, Libman and Sacks described valvular lesions in four patients with lupus and added nonrheumatic verrucous endocarditis to the syndrome complex of SLE [2]. Libman-Sacks valvular lesions are sterile fibrofibrinous vegetations that favor the left-sided heart valves and usually form on the ventricular surface of the mitral valve [3]. The disease progresses from a variable extent of inflammation along with fibrin deposits acutely to end stage or healed forms with a fibrous plaque. The pathogenesis is thought to involve the formation of fibrin-platelet thrombi, which organizes and leads to fibrosis and scarring with subsequent valve dysfunction [4]. Data comparing echocardiograph findings of 342 consecutive patients with SLE demonstrated that 11% of these patients had Libman-Sacks vegetations [5]. Those patients with LSE were likely to have had SLE longer with higher disease activity and more frequent episodes of pericarditis, lupus nephritis, and hemolytic anemia. Diverse and unusual initial presentation in lupus patients diagnosed with LSE has been reported in the literature [6], including an interesting case of cardiac arrest in a young lady as an initial manifestation of LSE [7]. This clinical scenario is unique as it is the first case to our knowledge reported in rheumatology literature in an adult patient in whom SLE was manifested initially as an embolic stroke secondary to LSE.

The modified Duke criteria utilizing pathologic and clinical criteria can be useful in helping differentiate between true infective endocarditis and LSE. Our patient only met one major criterion based on the echocardiograph findings. In addition infective endocarditis lesions are more likely to be located at the leaflet's line of closure, are homogeneous in echo reflectance, and may show a vibratory or rotary motion. In contrast, Libman-Sacks vegetations are usually located at the base, middle, or tip of leaflets, located on the atrial side of the mitral valve or vessel side of the aortic valve, and are of variable sizes and shapes and heterogeneous in echogenicity. The prevalence of Libman-Sacks vegetations is <10% by transthoracic echocardiogram and up to 30% by TEE. A recent case reported highlights the role of MRI in early recognition of LSE and differentiates between LSE and infective endocarditis [8].

On presentation, in our patient, there was a thought that this is a presentation of APS/CAPS given the positive lupus anticoagulant. When we applied revised Sapporo criteria, she did not meet criteria for definite APS, as only single reading of positive test was available to us at the time of presentation with vascular thrombosis. However, ongoing and cautious clinical and laboratory reassessment is required in individuals who do not meet diagnostic criteria for APS, particularly in patients with other “noncriteria” clinical or laboratory manifestations associated with but not specific for APS. Noncriteria clinical findings associated with aPL include heart valve disease, livedo reticularis, thrombocytopenia, nephropathy, and neurological manifestations. Noncriteria laboratory findings associated with APS include IgG or IgM aCL or anti-beta2-GPI levels in the range of 20 to 39 GPL or MPL units, IgA aCL and IgA anti-beta2-GPI, anti-phosphatidylserine and anti-phosphatidylethanolamine antibodies, anti-prothrombin antibodies, and antibodies to the phosphatidylserine-prothrombin complex. Our patient met noncriteria clinical findings for APS, including heart valve disease, thrombocytopenia, neurological manifestations, and borderline Cardiolipin IgG in addition to single reading of positive lupus anticoagulant.

Risks of valvular nodules, regurgitation, and verrucous endocarditis are more prevalent among patients with significantly elevated levels of anti-phospholipid antibodies (aPL). Many reports support an association. A meta-analysis of 23 primary studies, including 1656 SLE patients (668 with and 988 without aPL) and 508 cases of heart valvular disease, found a greater than threefold significantly elevated risk of valvular disease and 3.5-fold elevated risk of Libman-Sacks endocarditis among those with aPL, compared with those without aPL. The risk of valvular disease was the highest for lupus anticoagulant and IgG anticardiolipin antibodies [9].

The literature does not support the clear role of administration of glucocorticoid and/or cytotoxic therapy for valvular lesions, although there have been no trials. Antiplatelet or anticoagulation therapy should be considered for all patients with vegetation or significant valvular thickening and certainly for all patients with thromboembolic events. Our patient was started on anticoagulation due to her positive aPS antibody and embolic stroke. The suggested approach is similar to that in patients without valvular disease in the antiphospholipid syndrome. Whether anticoagulation should be recommended for patients with aPL-associated vegetation who have never had any thromboembolic event has not been well studied.

Surgical approach with valve replacement or valve repair may be necessary for some patients who develop severe mitral or aortic valve regurgitation or rarely for those with symptomatic stenotic lesions. Our patient underwent mitral valve repair due to severe mitral valve regurgitation.

## 3. Conclusion

In summary, this previously healthy young lady presented with acute vision changes and stroke like symptoms and was diagnosed with SLE, which was attributed to the following findings: thrombocytopenia, significant elevation of inflammatory markers, positive lupus serologies, and lupus anticoagulant antibodies. Brain imaging was consistent with an embolic stroke. Further workup, including echocardiography, supported the diagnosis of Libman-Sacks endocarditis in a patient with SLE. This case highlights the importance of early recognition of underlying connective tissue diseases with unusual initial presentation and timely management of these diseases in young patients.

## Figures and Tables

**Table 1:** Time course of laboratory data on initial presentation to ER, on presentation to our institution, and on discharge.

Laboratory	Reference range	Initial presentation to ER	Presentation at our institute	At the time of discharge
Sodium	135–145 mmol/L	135	135	140
Potassium	3.5–5.0 mmol/L	3.9	4.7	4.4
Chloride	98–111 mmol/L	100	104	108
Carbon dioxide	21–35 mmol/L	26	26	21
Blood urea nitrogen	10–15 mg/dL	23	30	23
Serum creatinine	<1.03 mg/dL	1.1	1.56	1.02
White blood cell	3.8–10.6 K/*μ*L	9.3	8.4	13.1
Hemoglobin	12.0–15.0 gm/dL	11.4	11.3	9.4
Platelet count	150–450 K/*μ*L	71	115	207
Absolute neutrophil count	1.80–7.70 K/*μ*L	NA	4.54	NA
Absolute lymphocyte count	1.10–4.00 K/*μ*L	NA	2.94	NA
Sedimentation rate	0–20 mm/hr	41	79	30

NA: not available.

## Abbreviations

LSE: Libman-Sacks endocarditis
SLE: Systemic lupus erythematous
aPS: Antiphospholipid syndrome.
